# Pre-ART Levels of Inflammation and Coagulation Markers Are Strong Predictors of Death in a South African Cohort with Advanced HIV Disease

**DOI:** 10.1371/journal.pone.0024243

**Published:** 2012-03-20

**Authors:** Lotty Ledwaba, Jorge A. Tavel, Paul Khabo, Patrick Maja, Jing Qin, Phumele Sangweni, Xiao Liu, Dean Follmann, Julia A. Metcalf, Susan Orsega, Beth Baseler, James D. Neaton, H. Clifford Lane, for the Project Phidisa Biomarkers Team

**Affiliations:** 1 Project Phidisa, Pretoria, South Africa; 2 National Institutes of Health, Bethesda, Maryland, United States of America; 3 Science Applications International Corporation, Frederick, Inc., Frederick, Maryland, United States of America; 4 University of Minnesota, Minneapolis, Minnesota, United States of America; National Institutes of Health, United States of America

## Abstract

**Background:**

Levels of high-sensitivity C-reactive protein (hsCRP), interleukin-6 (IL-6), and D-dimer predict mortality in HIV patients on antiretroviral therapy (ART) with relatively preserved CD4+ T cell counts. We hypothesized that elevated pre-ART levels of these markers among patients with advanced HIV would be associated with an increased risk of death following the initiation of ART.

**Methods:**

Pre-ART plasma from patients with advanced HIV in South Africa was used to measure hsCRP, IL-6 and D-dimer. Using a nested case-control study design, the biomarkers were measured for 187 deaths and two controls matched on age, sex, clinical site, follow-up time and CD4+ cell counts. Odds ratios were estimated using conditional logistic regression. In addition, for a random sample of 100 patients, biomarkers were measured at baseline and 6 months following randomization to determine whether ART altered their levels.

**Results:**

Median baseline biomarkers levels for cases and controls, respectively, were 11.25 vs. 3.6 mg/L for hsCRP, 1.41 vs. 0.98 mg/L for D-dimer, and 9.02 vs. 4.20 pg/mL for IL-6 (all p<0.0001). Adjusted odds ratios for the highest versus lowest quartile of baseline biomarker levels were 3.5 (95% CI: 1.9–6.7) for hsCRP, 2.6 (95%CI 1.4–4.9) for D-dimer, and 3.8 (95% CI: 1.8–7.8) for IL-6. These associations were stronger for deaths that occurred more proximal to the biomarker measurements. Levels of D-dimer and IL-6, but not hsCRP, were significantly lower at month 6 after commencing ART compared to baseline (p<0.0001).

**Conclusions:**

Among patients with advanced HIV disease, elevated pre-ART levels of hsCRP, IL-6 and D-dimer are strongly associated with early mortality after commencing ART. Elevated levels of inflammatory and coagulation biomarkers may identify patients who may benefit from aggressive clinical monitoring after commencing ART. Further investigation of strategies to reduce biomarkers of inflammation and coagulation in patients with advanced HIV disease is warranted.

**Trial Registration:**

Parent Study: ClinicalTrials.gov NCT00342355

## Introduction

An estimated 5.21 million people were living with HIV and AIDS in South Africa in 2009, more than in any other country [1]. It is estimated that in 2008 over 250,000 South Africans died of AIDS [2]. Multiple clinical trials have clearly demonstrated that combination antiretroviral therapy (ART) significantly reduces morbidity and mortality in HIV infected patients [3]–[14]. Fortunately, it is estimated that the number of persons initiating ART in sub-Saharan Africa has increased by nearly eight fold since 2004 [15]. However, HIV-infected patients in developing countries may have a higher mortality rate after commencing antiretroviral therapy compared to patients in developed countries [16], [17]. Most notably, studies conducted in sub-Saharan Africa demonstrate that mortality may be particularly high in the first three months after commencing ART [18]–[20]. There are likely to be a variety of causes for this increased risk, including immune reconstitution syndrome, opportunistic infections due to incomplete immune recovery, and toxicities associated with ART [21]–[23]. Predicting who has an increased short-term risk of mortality after starting ART could lead to modified clinical management or interventions that decrease mortality.

A nested case-control study from the clinical trial Strategies for Management of Antiretroviral Therapy (SMART) investigated the association of all-cause mortality and elevated levels of inflammatory and coagulation biomarkers in HIV-infected patients with CD4+ count >350 cells/mm^3^ [24], [25]. In this analysis from the SMART study, the majority of participants were on ART at baseline and most had HIV RNA levels ≤400 copies/mL. In this population, high sensitivity C-reactive protein (hsCRP) interleukin-6 (IL-6), and D-dimer measured at study entry were strongly related to all-cause mortality. These findings from SMART suggest that ongoing immune activation and disturbances in coagulation occur even during successful suppression of HIV replication. This may explain the findings from a growing body of literature demonstrating the increased risk of all-cause mortality and serious non-AIDS conditions such as cardiac, renal and hepatic disease in HIV-infected patients, even those with controlled viremia as compared to the general population [26]–[28].

Relatively little has been reported on the association of pre-ART levels of inflammation and coagulation markers with mortality in patients with advanced HIV disease [29]. The primary purpose of this investigation was to assess in an ART-naive group of patients with advanced HIV infection whether pre-ART levels of inflammatory and coagulation biomarkers are associated with mortality. In addition to those analyses, we also assessed whether initiation of ART lowered levels of these biomarkers, and compared pre-ART biomarker levels among patients with early versus late HIV infection, and HIV uninfected patients.

## Methods

### Study Population

**Figure 1:**
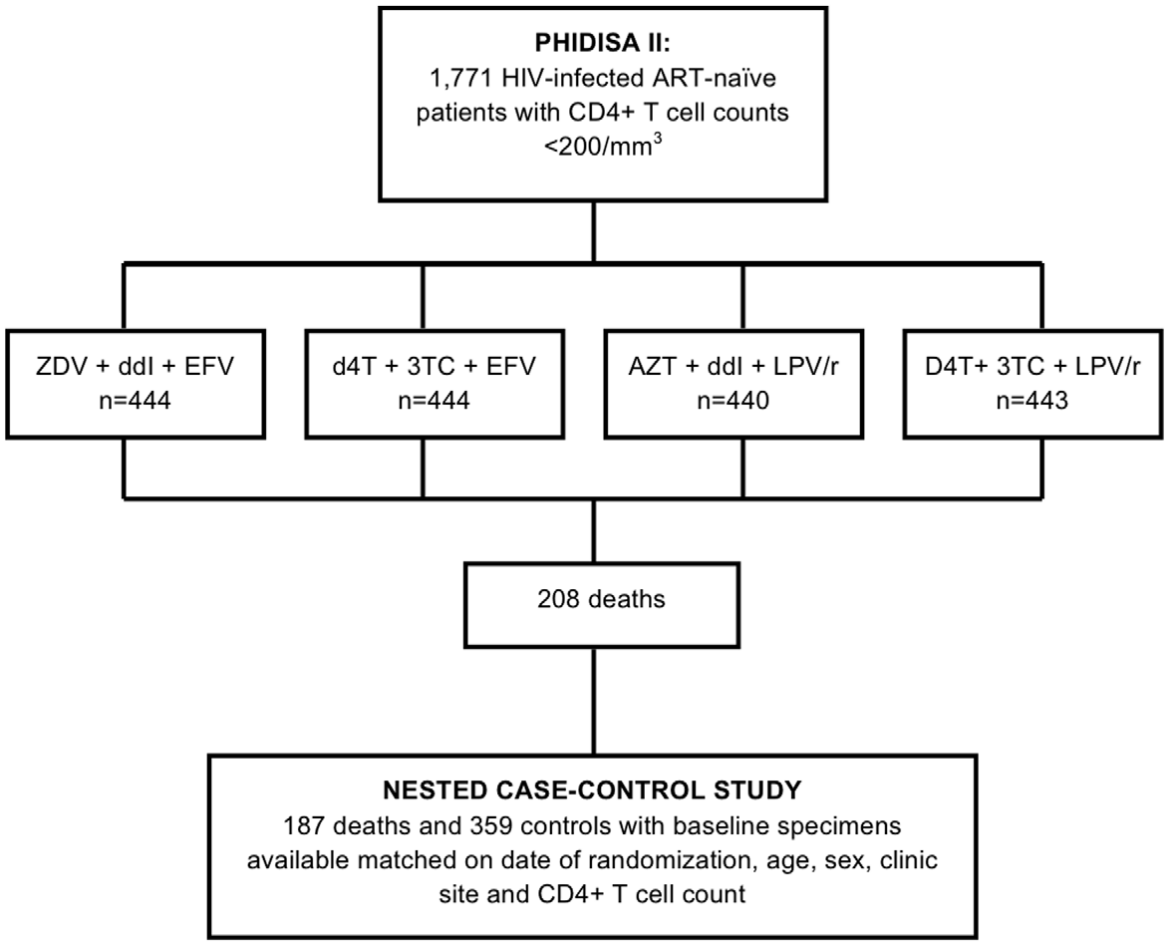
Phidisa II Study Design and Flow Diagram for Case-Control Substudy.

Phidisa II was an HIV treatment study that enrolled 1,771 antiretroviral naïve HIV-infected participants at six clinical sites in South Africa with CD4+ T cell counts <200 cells/mm^3^ (or <14% for patients post-splenectomy) and/or any history of or active AIDS-defining illness and with hemoglobin >9 g/dL (>8 g/dL for women), neutrophil count >500 cells/mm^3^, platelet count >250/uL, and serum liver transaminases below five times the upper limit of normal [30]. Following randomization to one of four initial ART regimens (figure 1), patients were seen monthly for three months, and then every three months for physical examination and laboratory studies, including measurement of CD4+ T cell count and HIV RNA level. Phidisa I was an epidemiologic study that followed HIV–uninfected individuals as well as HIV-infected participants with CD4 cell counts over 200 cells/mm^3^. Stored plasma specimens from participants in Phidisa I and Phidisa II were used for the analyses reported here.

The South African National Defence (SANDF) Force Ethics Committee and the National Institute of Allergy and Infectious Diseases Intramural Institutional Review Board approved the Phidisa I and Phidisa II protocols and their informed consent documents which contained language on the use of stored specimens as used in the current study. Written informed consent was obtained from all participants whose samples and data were used for this study.

### Laboratory Assays

Two inflammatory markers, hsCRP and IL-6, and one marker of coagulation, D-dimer, were prospectively chosen for these analyses since they have been associated with all-cause mortality in previous studies of patients with HIV infection. Assays for each of the three biomarkers evaluated in this study were performed at a single laboratory. Assays were performed using stored EDTA plasma from PPT tubes as follows: hsCRP assays [COBAS Integra (high sensitive); Roche; normal range <1.0 mg/L] were performed by Bio Analytical Research Corporation (BARC, Johannesburg, RSA); D-dimer assays (Innovance; Dade Behring Marburg GmbH; normal range <0.5 mg/L) were performed by Lancet Laboratories (Johannesburg, RSA); IL-6 assays (Quantikine HS; R&D Systems; normal range 0.428–8.870 pg/ml) were performed by Synexa (Cape Town, RSA). Hematology and serum chemistry assays, CD4+ T cell count, and HIV RNA were all measured by BARC.

### Biomarker Studies

Three related studies were performed. First, a nested case-control study of all-cause mortality was carried out to assess whether hsCRP, IL-6, and D-dimer, in pre-ART plasma were associated with mortality. Biomarker levels in each of the 187 patients who died in Phidisa II and who had baseline specimens stored were compared with two controls matched on age (+/−5 years), sex, follow-up time (+/− 3 months), clinic site (six sites) and CD4+ T cell count (+/− 25 cells/mm^3^). These variables were chosen to match cases and controls for the following reasons: age and CD4+ cell count were chosen because of their known association with mortality; sex was chosen to be consistent with other studies evaluating these biomarkers in HIV-infected patients; and site was chosen to control for potential variations in type of participant and nature of care. Second, to evaluate the effect of initiation of ART on markers of inflammation and coagulation, D-dimer, hsCRP and IL-6 were measured for a random sample of 100 study participants on plasma collected six months after commencing ART and compared with levels measured pre-ART. Third, since our cases and controls had advanced immunosuppression with CD4+ T cell counts below 200 cells/mm^3^, we compared their baseline biomarker levels to a group with early HIV infection (CD4+ cell counts of at least 200 cells/mm^3^) as well as an HIV negative group randomly drawn from participants in Phidisa I. For these analyses, D-dimer, hsCRP and IL-6 were determined for 80 HIV-infected patients with early HIV disease and 80 participants without HIV infection.

### Statistical Methods

Conditional logistic regression analyses for matched case-control studies were used to study the associations of baseline clinical variables and biomarkers with mortality. Biomarkers were categorized into quartiles and were also considered as continuous variables after log_10_ transformation. The following baseline covariates were included in adjusted (multivariate) regression analyses: HIV disease group (classified by history of tuberculosis, AIDS, or AIDS-related symptoms, hemoglobin (≤11.9 vs. >11.9 g/dL), platelet count (≤234 vs. >234/uL), aspartate aminotransferase (≤39.5 vs. >39.5 u/L) and white blood cell count (≤3.8 vs. >3.8×10^9^ cells/L). The above dichotomous variables were broken at the median. To determine whether associations with mortality varied for early and late deaths, the deaths and controls were divided in two groups based on the median survival time of the deaths (167 days). A Wald statistic was used to evaluate the effects of a biomarker as a function of death time. To assess the impact of ART on markers of coagulation and inflammation, D-dimer, hsCRP and IL-6 levels were compared at baseline and month six in a random sample of 100 patients. Analysis of covariance was used to determine if the six-month change (six month value minus baseline) differed between the randomized groups for continuous variables. A p-value less than 0.05 was considered as significant and all reported p-values are two-sided.

## Results

### Study Population

In Phidisa II, the average age of participants was 35 years and median CD4+ cell count at study entry for the overall Phidisa cohort was 106 cells/mm^3^. Over a median follow-up of 24.7 months 208 deaths occurred [30], of which 187 had baseline plasma specimens available for analysis. Of those 187 deaths, 172 had two matched controls and 15 had one matched control for a total of 359 controls. Table 1 shows the baseline characteristics for deaths and matched controls. There was no significant difference in baseline HIV load between cases and controls (p = 0.66). Median CD4+ cell count at study entry for the 187 deaths included in these analyses was 39 cells/mm^3^. Low body mass index (BMI), CD4+ T cell count, and hemoglobin, as well as elevated platelet count, aspartate aminotransferase (SGOT), and white blood cell count were associated with death (p<0.05).

**Table 1:** Median Baseline Characteristics of Deaths and Matched Controls.

	Deaths (n = 187)	Controls (n = 359)	p-value^a^
**Age** (25^th^, 75^th^ %ile)	35 (32, 39)	35 (32, 38)	NA
**Sex** (% female)	31	31	NA
**BMI** (kg/m^2^) (25^th^, 75^th^ %ile)	20.5 (18.3, 23.4)	22.3 (19.5, 25.7)	0.0001
**CD4+ count** (cells/mm^3^) (25^th^, 75^th^ %ile)	39 (9, 102)	42 (14, 110)	NA
**Log_10_ HIV RNA** (25^th^, 75^th^ %ile)	5.29 (4.92, 5.56)	5.25 (4.87, 5.61)	0.68
**Hepatitis B Surface Antigen** (% positive)	8.6	5.3	0.11
**Creatinine** (µmol/L) (25^th^, 75^th^ %ile)	70 (59, 86)	72 (61, 84)	0.18
**Hemoglobin** (g/dL) (25^th^, 75^th^ %ile)	11.2 (9.7, 12.5)	12.2 (11, 13.6)	<0.0001
**Platelet** (×10^9^/L) (25^th^, 75^th^ %ile)	264 (185, 334)	224 (174, 284)	0.0003
**SGOT** (IU/L) (25^th^, 75^th^ %ile)	42 (31, 60)	37 (29, 54)	0.04
**SGPT** (IU/L) (25^th^, 75^th^ %ile)	31 (22, 52)	29 (21, 44)	0.18
**WBC count** (×10^9^/L) (25^th^, 75^th^ %ile)	4.1 (3.1, 6.1)	3.6 (2.8, 4.7)	<0.0001

a p-value obtained from univariate conditional logistic model. NA = not applicable as characteristic was a matching factor.

### Baseline Biomarkers and All-Cause Mortality

Prior to starting ART, median hsCRP, D-dimer, and IL-6 levels were all significantly elevated in the deaths compared to controls (Table 2), and strong risk gradients for all-cause mortality were evident for higher levels of all three biomarkers (Table 3). Baseline levels of the three markers of inflammation and coagulation were directly correlated. The Spearman rank correlations for CRP vs. D-dimer was 0.353; CRP vs. IL-6 was 0.71; and D-dimer vs. IL-6 was 0.38 (all p<0.00001). A model with all three biomarkers was therefore considered. After adjusting for HIV disease state, hemoglobin, platelet count, aspartate aminotransferase, and white blood cell count, the odds ratios (OR) of the log_10_ transformed biomarkers associated with a one IQR higher level were: CRP HR 1.96 (p = 0.02); D-dimer HR 2.22 (p = 0.05); and IL-6 HR 1.12 (p = 0.57). Thus, baseline levels of CRP and D-dimer remained significantly correlated with mortality in this multivariate model.

**Table 2:** Baseline levels of hsCRP, IL-6 and D-dimer for Deaths and Matched Controls.

Biomarker	Deaths (N = 187) Median	Controls (N = 359) Median	Difference in log_10_ levels between Cases and Control	p-value^a^
	(25^th^, 75^th^ %ile)	(25^th^, 75^th^ %ile)	(SE)	
**hsCRP (ug/ml)**	11.25	3.60	0.30	<0.0001
	(2.90, 51.90)	(1.50, 11.30)	(0.07)	
**D-dimer (ug/ml)**	1.41	0.98	0.14	<0.0001
	(0.89, 2.82)	(0.65, 1.60)	(0.03)	
**IL-6 (pg/ml)**	9.02	4.20	0.22	<0.0001
	(3.24, 18.66)	(1.82, 8.13)	(0.05)	

a p-value obtained from a conditional logistic model with a single covariate corresponding to log_10_ transformed biomarker level.

**Table 3:** Risk of Death Associated with Baseline Biomarker Levels.

Biomarker	Type of analysis	<25th percentile (Reference)	25–49th percentile		50–74th Percentile		≥75th Percentile		OR associated with 1 IQR higher biomarker level	
			OR (95% CI)	p-value	OR (95% CI)	p-value	OR (95% CI)	p-value	OR (95% CI)	p-value
**hsCRP**	Univariate	1.0	1.2	0.64	2.0	0.02	4.9	<0.0001	3.0	*<0.0001*
			(0.6–2.1)		(1.1–3.4)		(2.8–8.5)		(2.2–4.2)	
	Adjusted	1.0	1.1	0.79	1.7	0.10	3.5	0.0001	2.5	*<0.0001*
			(0.5–2.2)		(0.9–3.1)		(1.9–6.7)		(1.7–3.6)	
**D-Dimer**	Univariate	1.0	1.4	0.26	2.2	0.008	3.8	<0.0001	5.8	*<0.0001*
			(0.8–2.5)		(1.1–3.8)		(2.2–6.6)		(3.1–10.8)	
	Adjusted	1.0	1.2	0.55	1.5	0.19	2.6	0.002	4.0	*<0.0001*
			(0.6–2.3)		(0.8–2.8)		(1.4–4.9)		(2.0–7.9)	
**IL-6**	Univariate	1.0	2.1	0.04	2.8	0.003	5.5	<0.0001	3.7	*<0.0001*
			(1.0–4.2)		(1.4–5.6)		(2.8–10.7)		(2.3–5.8)	
	Adjusted	1.0	1.7	0.14	1.9	0.08	3.8	0.0004	2.8	*<0.0001*
			(0.8–3.6)		(0.9–4.0)		(1.8–7.8)		(1.7–4.6)	

Odds ratios are derived from separate conditional logistic regression models for each biomarker (two unadjusted univariate and two adjusted models for each biomarker, one with quartiles and one with continuous level of biomarker after log_10_ transformation); adjusted models include HIV disease state, hemoglobin, platelet, aspartate aminotransferase, and white blood cell count (dichotomized at the median). Percentile cut-off points (IQR on log10 scale) are hsCRP: <1.80, 1.80–5.15, 5.15–20.05, ≥20.05 (0.36–1.31); D-dimer: <0.71, 0.71–1.11, 1.11–1.92, ≥1.92 (0.15–0.28); IL-6: <2.14, 2.14–4.92, 4.92–11.22, ≥11.22 (0.33–1.05).

Median baseline levels of all three biomarkers were significantly greater for the early deaths (defined as those deaths occurring before the median of all deaths: <67 days of commencing ART) compared to their respective controls: median baseline level of CRP was 29.4 for early deaths vs. 3.4 in their controls, D-dimer was 1.8 vs. 0.98, and IL-6 was 10.9 vs. 4.2 (all p<0.0001). Median baseline level of CRP was also significantly elevated for late deaths vs. their respective controls (6.7 vs. 3.8 respectively; p = 0.007), in contrast to those for D-dimer (1.2 vs. 1.0; p = 0.07) and IL-6 (5.0 vs. 4.0; p = 0.14) that were not significantly different. As shown in Table 4 and 5, after log_10_ transformation, a one interquartile range higher level of hsCRP was associated with an OR of 5.2 (95% CI 3.1–8.9) for early deaths versus 1.9 (1.2–2.8) for late deaths; a one interquartile range higher level D-dimer was associated with an OR of 14.0 (95% CI 5.1–38.3) for early deaths versus 2.5 (1.1–5.8) for late deaths; and a one interquartile range higher level of IL-6 was associated with an OR of 7.8 (95% CI 3.7–16.6) versus 1.7 (0.9–3.2) for late deaths. The interaction between biomarker levels and log_10_ death time of cases was significant for each marker (hsCRP, IL-6 and D-dimer) (p<0.01).

**Table 4:** Risk of Early Death Associated with Baseline Biomarker Levels.

Biomarker	Type of analysis	<25th percentile (Reference)	25–49th percentile		50–74th Percentile		≥75th Percentile		OR associated with 1 IQR higher biomarker level	
			OR	p-value	OR	p-value	OR	p-value	OR	p-value
			(95% CI)		(95% CI)		(95% CI)		(95% CI)	
**hsCRP**	Univariate	1.0	1.1	0.83	2.0	0.08	9.7	<0.0001	5.2	*<0.0001*
			(0.4–3.0)		(0.9–5.4)		(4.1–23.5)		(3.1–8.9)	
	Adjusted	1.0	1.1	0.93	1.8	0.29	5.8	0.001	4.2	*<0.0001*
			(0.3–3.3)		(0.7–4.9)		(2.0–16.5)		(2.2–7.9)	
**D-Dimer**	Univariate	1.0	1.3	0.59	3.2	0.009	6.9	<0.0001	14.0	*<0.0001*
			(0.5–3.5)		(1.3–7.6)		(2.9–16.4)		(5.1–38.3)	
	Adjusted	1.0	1.0 (0.4–2.9)	0.98	1.9 (0.7–5.0)	0.21	3.5 (1.3–8.9)	0.01	7.2	*<0.001*
									(2.5–21.1)	
**IL-6**	Univariate	1.0	7.9	0.01	10.7	0.002	28.7)	<0.0001	7.8	*<0.0001*
			(1.7–36.8)		(2.3–49.3)		(6.3–129.9)		(3.7–16.6)	
	Adjusted	1.0	6.1	0.03	6.4	0.02	15.8	0.0005	5.9	*<0.0001*
			(1.2–30.3)		(1.3–31.6)		(3.3–74.2)		(2.6–13.5)	

See Table 3, footnote.

**Table 5:** Risk of Late Death Associated with Baseline Biomarker Levels.

Biomarker	Type of analysis	<25th percentile (Reference)	25–49th percentile		50–74th Percentile		≥75th Percentile		OR associated with 1 IQR higher biomarker level	
			OR	p-value	OR	p-value	OR	p-value	OR	p-value
			(95% CI)		(95% CI)		(95% CI)		(95% CI)	
**hsCRP**	Univariate	1.0	1.2	0.70	2.0	0.08	2.5	0.02	1.9	*0.01*
			(0.5–2.5)		(0.9–4.1)		(1.2–5.2)		(1.2–2.8)	
	Adjusted	1.0	1.1	0.91	1.7	0.20	2.0	0.14	1.6	*0.07*
			(0.5–2.4)		(0.8–3.8)		(0.8–4.8)		(1.0–2.7)	
**D-Dimer**	Univariate	1.0	1.4	0.35	1.5	0.28	2.2	0.03	2.5	*0.04*
			(0.7–3.0)		(0.7–3.2)		(1.1–4.7)		(1.1–5.8)	
	Adjusted	1.0	1.5	0.32	1.3	0.52	2.3	0.07	2.4	*0.09*
			(0.7–3.4)		(0.6–3.2)		(0.9–5.5)		(0.9–6.3)	
**IL-6**	Univariate	1.0	1.1	0.80	1.6	0.30	1.8	0.17	1.7	*0.11*
			(0.5–2.7)		(0.7–3.8)		(0.8–4.4)		(0.9–3.2)	
	Adjusted	1.0	1.0	0.98	1.3	0.60	1.6	0.36	1.4	*0.32*
			(0.4–2.5)		(0.5–3.4)		(0.6–4.5)		(0.7–3.2)	

See Table 3, footnote 1.

### Differences in Biomarker Levels from Baseline to 6 Months after Commencing ART

In 100 randomly selected Phidisa II patients, mean log transformed biomarker levels at month 6 after commencing ART were significantly lower than baseline levels for D-dimer (−0.122; p<0.0001) and IL-6 (−0.274; p<0.0001) but not for hsCRP (−0.044, SD 0.649; p = 0.41). The changes did not differ by randomization assignment. No significant correlations were seen with change in CD4+ T cell count and changes in biomarker levels.

### Comparison of Baseline Biomarker Levels for HIV Uninfected Patients, and Patients with Early and Advanced HIV Infection

Table 6 shows that HIV uninfected participants had significantly lower baseline levels of all three biomarkers compared to patients with advanced HIV infection (p = 0.001 for hsCRP, p<0.0001 for D-dimer, and p = 0.01 for IL-6) after adjustment for baseline covariates. Compared to patients with advanced HIV infection, patients with early HIV infection had significantly lower baseline levels of hsCRP (p = 0.002) and D-dimer (p = 0.001), but they had significantly higher baseline levels of IL-6 (p = 0.01). Finally, compared to HIV-uninfected controls, baseline levels of D-dimer and IL-6, but not hsCRP, were significantly elevated in patients with early HIV infection (p = 0.003 for D-dimer, p<0.0001 for IL-6, p = 0.71 for hsCRP).

**Table 6:** Baseline Levels of hsCRP, IL-6 and D-dimer for HIV Uninfected Participants, and Participants with Early and Advanced HIV Infection.

	HIV uninfected (n = 80)	Early HIV infection* (n = 80)	Advanced HIV infection** (n = 100)
**Median Baseline Characteristics**			
**Age**	37	38	36
(25^th^, 75^th^ %ile)	(33, 42)	(35, 40)	(33, 39)
**Sex (% female)**	31	31	35
**CD4 + count (cells/mm3)**	NA	546	110
(25^th^, 75^th^ %ile)	NA	(409, 709)	(58, 175)
**HIV RNA**	NA	4,025	148,000
(25^th^, 75^th^ %ile)	NA	(1,036, 27,250)	(64,900, 304,000)
**Biomarker Levels** (median; 25^th^, 75^th^ %ile)			

	HIV uninfected	Early HIV infection* - No ART	Advanced HIV infection* - No ART	Intergroup Comparisons	
				Groups	p-value***
**hsCRP**	2.35 (0.9, 7.3)	3.05 (1.2, 6.2)	4.30 (1.95,12.3)	HIV negative vs. Early HIV	0.706
				HIV negative vs. Late HIV	0.001
				Early HIV vs. Late HIV	0.002
**D-Dimer**	0.52 (0.35, 0.7)	0.63 (0.43, 0.93)	0.86 (0.57,1.46)	HIV negative vs. Early HIV	0.003
				HIV negative vs. Late HIV	<0.0001
				Early HIV vs. Late HIV	0.001
**IL-6**	1.59 (1, 2.34)	4.99 (1.5, 12.5)	2.61 (1.42, 5.23)	HIV negative vs. Early HIV	<0.0001
				HIV negative vs. Late HIV	0.01
				Early HIV vs. Late HIV	0.01

NA = not applicable.

* CD4+ T cell count >350 cells/ml.

** CD4+ T cell count ≤200 cells/mL (or <14% for patients post-splenectomy) and/or any history of or current AIDS defining illness and with hemoglobin >9 g/dL (>8 g/dL for women), neutrophil count >500 cells/mL, platelet count >250/uL, and serum liver transaminases below five times the upper limit of normal.

*** Comparisons between the different groups are adjusted for age, sex and BMI.

## Discussion

This is the first study to demonstrate that markers of inflammation and coagulation measured immediately before ART is initiated are strongly associated with all-cause mortality in patients with advanced HIV-infection. In this case control study, patients who died after commencing ART had significantly elevated levels of D-dimers, hsCRP and IL-6 compared to matched controls, and strong risk gradients were observed for increasing levels of all three biomarkers. In addition, elevated baseline levels of inflammatory markers were more predictive of early mortality than late mortality after ART initiation. This association persisted in analyses that considered stage of HIV disease, hemoglobin, aspartate aminotransferase, platelet and white blood cell count. These findings suggest that HIV-induced activation of inflammatory and coagulation pathways in late-HIV infection have an adverse affect on mortality.

In our cohort of 100 random Phidisa II patients who initiated ART, IL-6 and D-dimer levels significantly decreased at month 6, whereas hsCRP did not. This association between IL-6 and D-dimer is consistent with other studies including HIV-infected as well as uninfected patients. [25], [29]–[33]. Also, whereas positive correlations were found with HIV viral load and hsCRP and D-dimer levels, a correlation did not exist between HIV viral load and IL-6 levels. This suggests that, in this population with advanced HIV infection, the pathway that leads to elevated IL-6 levels during HIV infection may be different than the pathway leading to elevations of other inflammatory biomarkers such as hsCRP. Other studies are needed to confirm this finding.

A study of inflammatory and coagulation biomarkers in HIV-infected patients in the SMART trial also demonstrated that baseline IL-6 and D-dimer levels were strongly related to all-cause mortality [24], [25]. Although the same biomarker assays were used in the SMART and our Phidisa II analyses, any comparison of biomarker levels between these studies must be made with a number of caveats: plasma from PPT was used in our study whereas plasma from EDTA tubes was used in SMART, the assays were performed at different laboratories, and there were differences in sample processing timelines (specimens in Phidisa II may have taken up to 24 hours to arrive in the laboratory for processing). With these caveats in mind, median baseline biomarker levels appear to be considerably higher in the Phidisa II mortality cases compared to those in SMART (hsCRP = 11.25 vs. 4.26 ug/ml; D-dimer = 1.41 vs. 0.49 ug/ml; IL-6 = 9.02 vs. 3.8 pg/ml, respectively). This is likely due to the fact that the patient populations enrolled in these studies were notably different. The SMART study population was relatively healthy and did not have advanced HIV disease; at baseline, the majority of participants were on ART and had a relatively high CD4+ T cell count [median = 545 (25^th^, 75^th^ percentile = 408, 693)]. In contrast, our study population was not on ART and had advanced HIV disease with low CD4+ T cell counts [median = 39 (25^th^, 75^th^ percentile = 9, 102)]. This supports the hypothesis that advanced immunosuppression in the context of unsuppressed HIV replication leads to an activation of inflammatory and coagulation pathways and, subsequently, increases mortality.

We also examined levels of hsCRP, IL-6 and D-dimer among HIV-uninfected patients, and patients with early and late HIV infection. In general, uninfected patients had lower levels of these biomarkers compared to patients with early disease, who in turn had lower levels compared to those patients with late disease, except for IL-6. This finding is consistent with other studies demonstrating that, compared with those who are HIV-uninfected, HIV-infected individuals have higher levels of inflammatory and coagulation markers [30].

A limitation to our study is that a definitive cause of death could not be established for all mortality cases in Phidisa II. It is known that 37 of the 208 deaths in Phidisa II followed a confirmed or probable AIDS event, and tuberculosis was the most common AIDS event observed in that trial. Nevertheless, analyses of the relationship between biomarker levels and cause of mortality could not be performed. Another limitation is that other potential confounding variables that may affect biomarker levels, such as smoking and hepatitis C status, were not collected and could not be adjusted for in our analyses.

In summary, among patients with advanced HIV disease, elevated pre-ART levels of hsCRP, IL-6 and D-dimer are strongly associated with early mortality after commencing ART. While the reasons for the increased risk are not fully understood, interventions other than ART that might decrease levels of inflammatory and coagulation markers warrant investigation. Until such interventions are found, more aggressive clinical monitoring may be warranted in patients with elevated biomarkers after commencing ART. Further studies are warranted to understand the underlying pathophysiology leading to these elevations in biomarker in order to develop interventions that may improve clinical outcomes in HIV infected patients starting ART.

## References

[1] UNAIDS Website.. http://www.unaids.org/en/KnowledgeCentre/HIVData/CountryProgress/2010CountryProgressAllCountries.asp.

[2] Lehohla PJ, Mid-year population estimates. Statistics South Africa (2009). http://www.statssa.gov.za/Publications/statsdownload.asp?PPN=P0302&SCH=4437.

[3] Palella FJ, Delaney KM, Moorman AC, Loveless MO, Fuhrer J (1998). Declining morbidity and mortality among patients with advanced human immunodeficiency virus infection. HIV Outpatient Study Investigators.. N Engl J Med.

[4] Michaels SH, Clark R, Kissinger P (1998). Declining morbidity and mortality among patients with advanced human immunodeficiency virus infection [Letter].. N Engl J Med.

[5] Detels R, Munoz A, McFarlane G, Kingsley LA, Margolick JB (1998). Effectiveness of potent antiretroviral therapy on time to AIDS and death in men with known HIV infections duration. Multicenter AIDS Cohort Study Investigators.. JAMA.

[6] Spira R, Marimoutou C, Binquet C, Lacoste D, Dabis F (1998). Rapid change in the use of antiretroviral agents and improvement in a population of HIV-infected patients: France, 1995–1997. Groupe d'Epidemiologie Clinique du SIDA en Aquitaine (GECSA).. J Acquir Immune Defic Syndr Hum Retrovirol.

[7] Correll PK, Law MG, McDonald AM, Cooper DA, Kaldor JM (1998). HIV disease progression in Australia in the time of combination antiretroviral therapies.. Med J Aust.

[8] Brodt HR, Kamps BS, Gute P, Knupp B, Staszewski (1997). Changing incidence of AIDS-defining illnesses in the era of antiretroviral combination therapy.. AIDS.

[9] Sendi PP, Bucher HC, Craig BA, Pfluger D, Battegay M (1999). Estimating AIDS-free survival in a severely immunosuppressed asymptomatic HIV-infected population in the era of antiretroviral triple combination therapy. Swiss HIV Cohort Study.. J Acquir Immune Defic Syndr.

[10] Gebhardt M, Rickenbach M, Egger M (1998). Impact of antiretroviral combination therapies on AIDS surveillance reports in Switzerland. Swiss HIV Cohort Study.. AIDS.

[11] Mocroft A, Vella S, Benfield TL, Chiesi A, Miller V (1998). Changing patterns of mortality across Europe in patients infected with HIV-1. EuroSIDA Study Group.. Lancet.

[12] Pezzotti P, Napoli PA, Acciai S, Boros S, Urciuoli R (1999). Increasing survival time after AIDS in Italy: the role of new combination antiretroviral therapies. Tuscany AIDS Study Group.. AIDS.

[13] Walsh JC, Jones CD, Barnes EA, Gazzard BG, Mitchell SM (1998). Increasing survival in AIDS patients with cytomegalovirus retinitis treated with combination antiretroviral therapy including HIV protease inhibitors.. AIDS.

[14] Clifford DB, Yiannoutsos C, Glicksman M, Simpson DM, Singer EJ (1999). HAART improves prognosis in HIV-associated progressive multifocal leukoencephalopathy.. Neurology.

[15] World Health Organization, ‘Universal Access by 2010’, Available: http://www.who.int/hiv/data/en/. Accessed 2011 Apr 1

[16] Egger M for The Antiretroviral Therapy in Lower Income Countries (ART-LINC) Collaboration and ART Cohort Collaboration (ART-CC) Groups (2006). Mortality of HIV-1 infected patients in the first year of antiretroviral therapy: comparison between low-income and high-income countries.. Lancet.

[17] Marazzi MC, Liotta G, Germano P, Guidotti G, Altan AD (2008). Excessive early mortality in the first year of treatment in HIV type 1-infected patients initiating antiretroviral therapy in resource-limited settings.. AIDS Res Hum Retroviruses.

[18] Johanessen A, Naman E, Ngowi BJ, Sandvik L, Matee MI (2008). Predictors of mortality in HIV-infected patients starting antiretroviral therapy in a rural hospital in Tanzania.. BMC Infectious Disease.

[19] Stringer JS, Zulu I, Levy J, Stringer EM, Mwango A (2006). Rapid scale-up of antiretroviral therapy at primary care sites in Zambia: feasibility and early outcomes.. JAMA.

[20] Lawn SD, Myer L, Orrell C, Bekker LG, Wood R (2005). Early mortality among adults accessing a community-based antiretroviral service in South Africa: implications for programme design.. AIDS.

[21] Etard JF, Ndiaye I, Thierry-Mieg M, Gueye NF, Gueye PM (2006). Mortality and causes of death in adults receiving highly active antiretroviral therapy in Senegal: a 7-year cohort study.. AIDS.

[22] Egger M, May M, Chene G, Phillips AN, Ledergerber B (2002). Prognosis of HIV-infected patients starting highly active antiretroviral therapy: a collaborative analysis of prospective studies.. Lancet.

[23] Palella FJ, Baker R, Moorman AC, Chmiel JS, Wood KC (2006). Mortality in the Highly Active Antiretroviral Therapy Era: Changing Causes of Death and Disease in the HIV Outpatient Study.. J Acquired Immun Defic Syndr.

[24] El-Sadar WM, Grund B, Neuhaus J, Babiker A, Cohen CJ (2006). CD4 count guided interruption of antiretroviral treatment.. NEJM.

[25] Kuller LH, Tracy R, Belloso W, De Wit S, Drummond F (2008). Inflammatory and Coagulation Biomarkers and Mortality in Patients with HIV infection.. PloS Medicine.

[26] Neuhaus J, Angus B, Kowalska JD, La Rosa A, Sampson J (2010). Risk for all-cause mortality associated with nonfatal AIDS and serious non-AIDS events among adults infected with HIV.. AIDS.

[27] Phillips AN, Neaton J, Lundgren JD (2008). The role of HIV in serious diseases other than AIDS.. AIDS.

[28] Morcroft A, Reiss P, Gasoirowski J, Ledergerber B, Chiesi A (2009). Serious fatal and non fatal non-AIDS defining illnesses in Europe..

[29] Jong E, Louw S, van Gorp E, Meijers J, ten Cate H (2010). The effect of initiating combined antiretroviral therapy on endothelial cell activation and coagulation markers in South African HIV-infected individuals.. Thrombosis and Haemostasis.

[30] Ratsela A, for the Phidisa 2 Study Group (2010). A randomized factorial trial comparing 4 treatment regimens in treatment-naïve HIV-infected persons with AIDS and/or <200 CD4+ T cells/mm^3^ in South Africa.. J Infect Dis.

[31] Neuhaus J, Jacobs DR, Baker JV, Calmy A, Duprez D (2010). Markers of inflammation, coagulation, and renal function are elevated in adults with HIV infection.. J Infect Dis.

[32] Cohen HJ, Harris T, Piper CF (2003). Coagulation and activation of inflammatory pathways in the development of function decline and mortality in the elderly.. Am J Med.

[33] Shorr AF, Thomas SJ, Alkins SA, Fitzpatrick TM, Ling GS (2002). D-dimer correlates with proinflammatory cytokine levels and outcomes in critically ill patients.. Chest.

